# Impact of a Booklet about Diabetes Genetic Susceptibility and Its Prevention on Attitudes towards Prevention and Perceived Behavioral Change in Patients with Type 2 Diabetes and Their Offspring

**DOI:** 10.4061/2011/365132

**Published:** 2010-10-13

**Authors:** Masakazu Nishigaki, Eiko Sato, Ryota Ochiai, Taiga Shibayama, Keiko Kazuma

**Affiliations:** ^1^Department of Adult Nursing, Graduate School of Medicine, University of Tokyo, 7-3-1, Hongo, Bunkyo-ku, Tokyo, Japan; ^2^Department of Nursing, Faculty of Health Care, Kiryu University, Gunma, Japan; ^3^Graduate School of Comprehensive Human Sciences, Institute of Nursing Sciences, University of Tsukuba, Ibaraki, Japan

## Abstract

*Background*. Offspring of type 2 diabetic patients are at a high risk of type 2 diabetes. Information on diabetes genetic susceptibility and prevention should be supplied to the offspring. *Methods*. A six-page booklet on diabetes genetic susceptibility and prevention was distributed to 173 patients who ere ordered to hand it to their offspring. The patients answered a self-administered questionnaire on booklet delivery and attitudinal and behavioral changes toward diabetes and its prevention in themselves and their offspring. *Results*. Valid responses were obtained from 130 patients. Forty-nine patients had actually handed the booklet. Booklet induces more relief than anxiety. From the patient's view, favorable attitudinal and/or behavioral changes occurred in more than half of the offspring who were delivered the booklet. *Conclusion*. The booklet worked effectively on attitudes and behaviors toward diabetes and its prevention both in patients and their offspring. However, the effectiveness of patients as information deliverers was limited.

## 1. Introduction

Type 2 diabetes is a global burden and its etiology is a complex interaction of genetic and environmental factors. Individuals genetically predisposed to type 2 diabetes x are thus important targets for preventive strategies. Genetic understanding of diabetes has drastically progressed through the use of exhaustive methods for searching candidate genes, such as the genome-wide assay [1]. Many investigators have found candidate genes for type 2 diabetes, and most of them show a 1.4-fold increase in individual risk of type 2 diabetes [2]. Recent studies have shown that genotype adds slightly more information to predictive models which consist of common risk factors, including family history [3–6]. So genetic screening using information on individual genetic variants will become technically possible in future, but its usefulness as a predictive factor is still insufficient. In addition, translational research in the field of genetic screening and the discussion about ethical, legal, and social issues are now lagging behind the progress of technology [7, 8].

Relatives of type 2 diabetic patients show a higher risk of developing type 2 diabetes epidemiologically since they are likely to share genetic predispositions and have lifestyle habits similar to those of their parents [9]. In particular, individuals with an affected first-degree relative display a 2.3–5.5-fold higher risk of type 2 diabetes, independent of sex, age, race/ethnicity, body mass index (BMI), and other demographic characteristics [10]. Therefore, family history has been used to screen high-risk populations [11–13]. Thus, family history is still an important tool for identification of high-risk populations [14, 15].

Intervention should be based on established preventive strategies for other high-risk populations, such as obese or subjects with impaired glucose tolerance [16, 17]. These preventive strategies were conducted in face-to-face personalized form, and previous intervention study for offspring of type 2 diabetes patients in such form revealed significant effect on lifestyle change [18, 19]. However, community settings are more complex and have fewer resources compared with research settings. Direct intervention by medical professionals to communicate with healthy offspring is especially difficult because of lack of opportunities of communication [20]. Thus, some surrogate strategy is required to provide the prevention strategy to offspring.

For high-risk individuals to become actively involved in prevention, recognition of the risk for the disease is crucial [21, 22]. In some diseases with known genetic susceptibility, affected relatives can play effective roles in promoting adoption of preventive behavior to other unaffected family members [23, 24]. But for diabetes, previous studies have shown that although patients recognize the necessity of advising their offspring to adopt preventive behavior, they do not necessarily advise their offspring due to underestimated risk perception [25]. Moreover, preventive behavior in offspring may not be necessarily facilitated even if their parents advise them [26]. This ineffectiveness is mainly due to unmet needs for information source: offspring of type 2 diabetic patients want information about disease susceptibility and prevention directly from medical professionals [26]. From these perspectives, tool development which enables patients to deliver information on diabetes genetic susceptibility and prevention made by medical professional to their offspring is necessary.

This study aimed to develop a delivery tool that consists of information on genetic susceptibility to diabetes and diabetes prevention and to investigate its effect on attitudes towards prevention and perceived behavioral change in both patients and their offspring.

## 2. Material and Methods

### 2.1. Subjects

Subjects of this study were type 2 diabetic outpatients who were treated at a general hospital with more than 450 beds in a major urban area of Gunma Prefecture, Japan. Patient inclusion criteria were as follows: diagnosed with type 2 diabetes for a minimum of three months prior to study start, aged between 20 and 70, and have offspring. Patients who had serious mental illness, or other functional impairments making them unable to answer questionnaires were excluded. Patients with the following severe impairment due to diabetes complications were also excluded: renal failure requiring dialysis, amputation of lower limbs, or blindness.

### 2.2. Information Delivery Tool

A six-page booklet on disease susceptibility, focusing especially on genetic predisposition and prevention of disease onset, was prepared. The booklet was drafted based on the traditional health belief model (Table 1, details were shown in Table 5) [22]. The draft contents of the booklet were developed based on a review of existing literatures. Expert opinions from physicians specializing in diabetes and genetics and from certified genetic counselors were taken into account throughout the process of developing the booklet, including confirmation of content validity. The face validity of the booklet was also confirmed by a few patients and offspring of a type 2 diabetes patient.

### 2.3. Research Procedure

After obtaining written consent, the patients were asked to complete a questionnaire for initial data collection. The booklet was then given to the patients with an attached letter in December 2009. The patients were asked to read the booklet carefully and to hand it to their offspring based on their autonomy. Two months after providing the booklet, a questionnaire that aimed to investigate outcome measures was sent via mail to the patients. The questionnaire was returned to the researchers via mail.

### 2.4. Outcome Measures

During initial data collection, information on patients' age, sex, insulin use, diabetes complications, and glycemic control (HbA1c) was collected from medical records. The questionnaire had questions on years since diagnosis, height, weight, educational background, and occupational status.

To determine outcome measures, the patients were questioned about the delivery status of the booklet and its effect on them and their offspring. First, the patients were asked about changes in their attitude toward diabetes risk and its prevention in their offspring after reading the booklet, and these were assessed based on five parameters, namely, *anxiety*, *relief*, *sense of deliverer*, *zeal to know more about heritability of diabetes,* and *sense of role model, *which were judged based on the following options presented to the patients in a questionnaire: (1) The worry about my offspring getting diabetes has increased; (2) I feel comforted because it turned out that diabetes can be prevented; (3) I should share my knowledge about diabetes with my offspring; (4) I found that I want to know more about heritability of diabetes; (5) I should be a model for my offspring for dietary and exercise habits. Each parameter was rated on a 5-point Likert scale (from 1 = strongly disagree to 5 = strongly agree). Then, the patients were also questioned whether they had handed the booklet to their offspring. They had three options for the answer in the questionnaire: (1) I have already handed it; (2) Although I have not handed it yet, I intend to hand it; (3) I have not handed it, and I don't intend to hand it. The patients who answered that he/she had already handed the booklet to their offspring were then questioned about the change in their offspring after receiving the booklet, and this was assessed based on seven parameters, namely, *anxiety*, *relief*, *attitude toward diabetes*, *dietary behavior change*, *physical activity change*, *attitude toward parent,* and *indifference,* which were judged based on the following options presented to the patients in a questionnaire: (1) The worry about diabetes has increased; (2) He/She feels comforted because it turned out that diabetes can be prevented; (3) He/She pays more attention to diabetes than previously; (4) He/She began to intentionally follow a low-fat and fiber-rich diet; (5) He/She began to exercise regularly; (6) He/She came to ask me about diabetes; (7) He/She showed hardly any interest. Each parameter was rated on a 5-point Likert scale (from 1 = strongly disagree to 5 = strongly agree). Face validity of questionnaire and booklet was confirmed through the pre-test on five offspring whose parent was diabetic patient.

### 2.5. Data Analysis

First, descriptive statistics for basic characteristics and outcome measures were tabulated. Then, the relationship between delivery status and other measures was determined to clarify which factors would facilitate information delivery using the chi-square test or Student's *t*-test. As we aimed to detect tendencies in intrasubject priority placed on psychological effects, the agreement score difference between relief and anxiety was calculated in each patient, and Wilcoxon signed rank sum test was performed. The SAS version 9.13 software (SAS Institute, Cary, NC, USA) was used for statistical analysis, and the significance level was set at *P* < .05.

Study protocols were approved by the Institutional Review Board of the Graduate School of Medicine, University of Tokyo, and written informed consent was obtained before patient enrollment.

## 3. Results

Among 173 patients who were eligible and who consented to participate in this study, a valid response was obtained only from 130 patients (75.1%).  Table 2 shows patient characteristics: male 57.7% (*n* = 75), mean age 59.9 years (SD = 7.4), and 62.3% patients living with their offspring. The patient disease status was as follows: mean HbA1c 7.2% (SD = 0.9), mean BMI 25.7 (SD = 4.9), one diabetic complication being observed in 15.4%–36.9% of the patients.

Changes in patients' attitude toward diabetes risk and its prevention in their offspring are shown in Table 3. Increased worry about diabetes occurrence in the offspring was observed in 61.7% of the patients, but comparatively more patients reported increased relief (76.9%). Actually, 56 (43.1%) patients rated higher agreement for the question about relief than the question about anxiety, while 54 (41.5%) rated equally and 19 (14.6%) rated higher for anxiety (*P* < .0001, Wilcoxon signed rank test). Similarly, more than 70% of the patients reported a favorable change in the other three parameters.

Forty-nine patients (37.7%) had already handed the booklet to their offspring, and sixty-three patients (48.5%) expressed their intention to deliver. Fifteen patients (11.5%) refused to deliver the booklet. The patients who lived with their offspring tended to deliver the booklet more frequently to their offspring (*P* = .01, chi-square test). No other patient characteristics showed a significant relationship with delivery status.

Forty-nine patients who had already handed the booklet to their offspring were questioned about observed changes in their offspring after receiving the booklet (Table 4). Twenty-one (42.4%) patients reported that worry about diabetes occurrence seemed to have been increased in their offspring, but comparatively more patients reported increased relief (57.1%). Actually, 18 (36.7%) patients rated higher agreement for the question about their offspring's relief than the question about anxiety, while 19 (38.8%) rated equally and 10 (20.4%) rated higher for anxiety (*P* < .04, Wilcoxon signed rank test). Approximately, 70% of the patients who handed the booklet to their offspring thought that their offspring began paying more attention to diabetes. About 60% of patients thought that their offspring had changed their dietary habits, while less than half (44.9%) of them thought that their offspring had changed their physical activity habits (*P* = .046, Wilcoxon signed rank test). Nineteen (38.8%) patients reported that their offspring offered information on diabetes to them, while 13 (26.5%) regarded their offspring as indifferent to the booklet.

## 4. Discussion

The present study investigated utilization of type 2 diabetic patients as information deliverers and the effect of an information delivery tool generated by medical professionals on attitudes towards prevention and perceived behavioral change.

Information on disease susceptibility would sometimes be a psychological burden for high-risk people, especially to those who are genetically predisposed [31]. But some previous research has indicated that such information has a positive effect on psychological factors in diabetes [7, 32]. Consistent with these studies, both patients and their offspring in this study reported stronger agreement to increased relief than increased anxiety. Active utilization of genetic information should be considered in case of preventable or curable diseases. However, we must not ignore the fact that 14.6% of patients and 20.4% of offspring felt more anxiety than relief. Healthcare providers must consider the negative impact of information disclosure about genetic disease predisposition. So, establishment of an infrastructure to consider ethical, legal, and social issues is necessary for utilization of genetic information, even if the beneficial effects of genetic information exceed its detrimental effects generally.

The information supplied in this study produced favorable attitudinal and behavioral changes toward diabetes risk and its prevention. The patients began to pay more attention to diabetes prevention in their offspring and to be aware of their role as information deliverers and role models with respect to lifestyle. Offspring who wanted information to be supplied by medical professionals [26] also revealed favorable attitudinal and/or behavioral changes as reported by the patients. More than half of patients who handed booklet to their offspring reported that their offspring changed their dietary habits. Exercise habits changed less frequently than dietary habits, which may show that more barriers exist to engaging in regular exercise [26, 33]. A previous study has shown that advice from parents would not produce behavioral changes in the offspring because they want information directly from medical professionals [26]. In this research, information supplied from a medical professional in booklet form stimulated preventive behavior among offspring of type 2 diabetic patients. On the other hand, from the viewpoint of information delivery, a different systematic strategy is needed to facilitate patient's actions because booklet delivery was delayed in about half of all the subjects, although most intended to deliver booklet. A separate living arrangement is an obvious barrier to information delivery. A possible strategy for delivering the booklet without failure is to mail it directly to offspring. However, such a strategy involves ethical issues, because it is crucial to collect personal information (i.e., a mailing address) for this purpose. Further consideration is needed to establish an ethically sound and reliable strategy for information delivery.

### 4.1. Limitations

First, this study obtained information on attitudinal and behavioral changes in the offspring through their parents and not directly through their offspring. This would surely be a source of bias; however, whether it would contribute to an optimistic or to a pessimistic bias remains unclear.

In addition, whether these findings represent a specific characteristic of diabetic patients and their offspring is unclear because this study did not contain a control group of nondiabetic adult patients. Careful consideration is necessary because the current study was conducted in a single institutional setting and not in randomly sampled subjects. In addition, Table 1 shows that subjects of this study were an approximately fair-controlled (7.1%, HbA1c) population. This point would weaken the ability to extrapolate the results of this study. The nature of this cross-sectional study also weakens its validity. Although this study focused on “change” caused by the booklet and stated it clearly in the questionnaire, the subjects might have not been able to distinguish between “change” and “actual status.” A further prospective interventional study conducted on offspring directly with an adequate control group is needed to clarify the effect of information delivery on preventive behavior among offspring. Finally, results were limited by the self-reported nature of the questionnaire. Subjects answers were based on subjective perception. In particular, questions about preventive behaviors were influenced by the subjective nature of the questionnaire. More objective questions based on quantitative data of real activity such as total calorie intake or duration of exercise should be obtained to clarify the results of this study.

In conclusion, this research showed that information supplied from a medical professional could effectively stimulate preventive behavior among offspring of type 2 diabetic patients. On the other hand, utilization of the patients as information deliverers has some limitation in its current form. Thus, development of a different strategy for direct delivery of information to offspring of type 2 diabetic patients is necessary.

## Figures and Tables

**Table 1:** Contents of the booklet.

Page	Core elements of HBM	Contents
1	Perceived seriousness	Information on symptoms and complications related to diabetes.
2	Perceived susceptibility	Drastic increase in the number of diabetic patients, and implications on genetic-environmental interaction. Causes of diabetes, such as genetic predisposition, high-fat meal, and/or sedentary lifestyle [27, 28].
3	Perceived susceptibility	Information on genetic predisposition, decreased insulin secretion, and decreased insulin sensitivity easily caused by high-fat meal. [15]
4	Perceived susceptibility	Individuals with an affected first-degree relative display a 2.3–5.5-fold higher risk of type 2 diabetes [10] since such individuals seem to have similar genetic predisposition and lifestyle as those of the patients.
5	Perceived benefits	The risk of acquiring diabetes can be modified by having a low-fat diet and by increasing physical activity [29, 30].
6	Perceived barriers	Abstract of concrete methods to modify diet and physical activity and recommendation to refer professionals for individualized prevention [29, 30].

HBM: Health Belief Model [22].

**Table 2:** Patient characteristics (*N* = 130).

	*n* (%) or mean ± SD
Male	75 (57.7)
Age	59.9 ± 7.4
Living with their offspring	81 (62.3)
Current occupation	
Full time	64 (49.2)
Part time	20 (15.4)
Homemaker	22 (16.9)
Inoccupation	24 (18.5)
Educational status	
High school or less	100 (76.9)
Beyond high school	30 (23.1)
Current diabetes medication	
No medication	13 (10.0)
Taking oral medication	76 (58.5)
Taking insulin	41 (31.5)
BMI	25.7 ± 4.9
Years since diagnosis	11.2 ± 7.8
HbA1c	7.2 ± 0.9
Current diabetes complication	
Nephropathy	41 (31.5)
Neuropathy	20 (15.4)
Retinopathy	48 (36.9)

**Table 3:** Change in patients' attitude toward diabetes risk and its prevention in their offspring *N* = 130.

Items	Strongly agree	Agree	Neutral	Disagree	Strongly disagree
	*n* (%)	*n* (%)	*n* (%)	*n* (%)	*n* (%)
The worry about my offspring getting diabetes has increased.	28 (21.5)	51 (39.2)	36 (27.7)	11 (8.5)	4 (3.1)
I feel comforted because it turned out that diabetes can be prevented.	52 (40.0)	48 (36.9)	21 (16.2)	7 (5.4)	1 (0.8)
I should share my knowledge about diabetes with my offspring.	57 (43.8)	47 (36.2)	19 (14.6)	6 (4.6)	1 (0.8)
I found that I want to know more about heritability of diabetes.	55 (42.3)	42 (32.3)	26 (20.0)	5 (3.8)	2 (1.5)
I should be a model for my offspring for dietary and exercise habits.	71 (54.6)	36 (27.7)	16 (12.3)	6 (4.6)	1 (0.8)

**Table 4:** Patient-regarded change in offsprings' attitude toward diabetes risk and its prevention *N* = 49.

Items	Strongly Agree	Agree	Neutral	Disagree	Strongly disagree
	*n* (%)	*n* (%)	*n* (%)	*n* (%)	*n* (%)
The worry about diabetes has increased.	10 (20.4)	11 (22.4)	17 (34.7)	5 (10.2)	5 (10.2)
He/She feels comforted because it turned out that diabetes can be prevented.	13 (26.5)	15 (30.6)	15 (30.6)	2 (4.1)	2 (4.1)
He/She pays more attention to diabetes than previously.	14 (28.6)	15 (30.6)	16 (32.7)	1 (2.0)	2 (4.1)
He/She began to intentionally follow a low-fat and fiber-rich diet.	11 (22.4)	18 (36.7)	13 (26.5)	3 (6.1)	3 (6.1)
He/She began to exercise regularly.	10 (20.4)	12 (24.5)	18 (36.7)	4 (8.2)	5 (10.2)
He/She came to ask to me about diabetes.	8 (16.3)	11 (22.4)	17 (34.7)	5 (10.2)	7 (14.3)
He/She showed hardly any interest.	6 (12.2)	7 (14.3)	17 (34.7)	10 (20.4)	8 (16.3)

**Table 5:** Details of a booklet about diabetes prevention for people with a family history of diabetes.

Page	*Core elements of Health Belief Model* and component details
1	*Perceived Seriousness * Type 2 diabetes is an illness that shows a high blood glucose level. Blood glucose level is raised by defects in the ability of insulin secretion and/or use of insulin. Diabetes is a cause of serious complications: eye complications (sometimes causes blindness), nephropathy (sometimes requires dialysis), and neuropathy (sometimes requires foot amputation). Diabetes is a significant risk factor of critical macrovascular complications such as stroke or myocardial infarction.

2	*Perceived Susceptibility * Diabetes is one of the most common diseases in Japan. The estimated number of possible/probable diabetes patients was 22 million in 2007, which is a 1.6-fold increase compared to 10 years ago (13.7 million). Such rapid increase in the number of diabetes patients is mainly due to lifestyle changes in the past decades: physical activity became considerably less due to technological progress in transportation and/or automation; dietary habits have changed. The usual meal consists of more fat and less fiber compared to a traditional Japanese meal. In addition, there is genetic susceptibility that of being unable to adjust to environmental change.

3	*Perceived Susceptibility * Genetic predispositions are mainly characterized by two biological features: decreased insulin secretion, and decreased insulin sensitivity easily caused by high-fat meals. People with such a predisposition are more prone to experiencing insulin resistance and hyperinsulinemia, which would connect to beta cell function loss. These predispositions would be characterized by variants in disease susceptible gene. The vast of recent research try to explicate genetics in diabetes, but its application to preventive medicine still remains under development.

4	*Perceived Susceptibility * On the other hand, an epidemiological study has clearly shown that individuals with an affected first-degree relative display a 2.3–5.5-fold higher risk of type 2 diabetes. First-degree relatives share half of their genes. Moreover, the family (not limited to first degree relative) may share a similar lifestyle to that of the patients. Thus family history is a significant risk factor for diabetes from the viewpoint of both environmental/genetic predisposition.

5	*Perceived benefits * Although family history indicates a possible genetic predisposition, which cannot be modified, this does not imply a definite future occurrence of diabetes. Since diabetes occurs due to a complicated interaction between genetic/environmental factors, you can act against diabetes by modifying your lifestyle even if you have a genetic predisposition. For example: adequate energy intake that meets the low ability of insulin secretion would protect your beta cell function; adequate calorie intake and low-fat meals would prevent the development of insulin resistance; regular physical activity is useful for controlling energy consumption and increasing insulin sensitivity.

6	*Perceived barriers * Lifestyle modification is useful in diabetes prevention. However, a concrete method of modifying one's lifestyle is not necessarily understood. A summary of concrete methods of modifying diet and physical activity is shown (e.g., low-fat, high-fiber meals and regular exercise (3 or more days/week and over 30 minutes/each time)). It is important to take professional advice to find the most effective treatment and tailor preventive behavior to your lifestyle. Health professionals can help you learn how to integrate a favorable diet and/or physical activity into your daily life.
